# Mechanism of Virus Inactivation by Cold Atmospheric-Pressure Plasma and Plasma-Activated Water

**DOI:** 10.1128/AEM.00726-18

**Published:** 2018-08-17

**Authors:** Li Guo, Ruobing Xu, Lu Gou, Zhichao Liu, Yiming Zhao, Dingxin Liu, Lei Zhang, Hailan Chen, Michael G. Kong

**Affiliations:** a State Key Laboratory of Electrical Insulation and Power Equipment, Center for Plasma Biomedicine, Xi'an Jiaotong University, Xi'an, People's Republic of China; b School of Life Science and Technology, Xi'an Jiaotong University, Xi'an, People's Republic of China; c Department of Applied Physics, School of Science, Xi'an Jiaotong University, Xi'an, People's Republic of China; d Frank Reidy Center for Bioelectrics, Old Dominion University, Norfolk, Virginia, USA; e Department of Electrical and Computer Engineering, Old Dominion University, Norfolk, Virginia, USA; Centers for Disease Control and Prevention

**Keywords:** virus, cold atmospheric-pressure plasma, plasma-activated water, bacteriophage, reactive oxygen species

## Abstract

Viruses cause serious pathogenic contamination that severely affects the environment and human health. Cold atmospheric-pressure plasma efficiently inactivates pathogenic bacteria; however, the mechanism of virus inactivation by plasma is not fully understood. In this study, surface plasma in argon mixed with 1% air and plasma-activated water was used to treat water containing bacteriophages. Both agents efficiently inactivated bacteriophages T4, Φ174, and MS2 in a time-dependent manner. Prolonged storage had marginal effects on the antiviral activity of plasma-activated water. DNA and protein analysis revealed that the reactive species generated by plasma damaged both nucleic acids and proteins, consistent with the morphological examination showing that plasma treatment caused the aggregation of bacteriophages. The inactivation of bacteriophages was alleviated by the singlet oxygen scavengers, demonstrating that singlet oxygen played a primary role in this process. Our findings provide a potentially effective disinfecting strategy to combat the environmental viruses using cold atmospheric-pressure plasma and plasma-activated water.

**IMPORTANCE** Contamination with pathogenic and infectious viruses severely threatens human health and animal husbandry. Current methods for disinfection have different disadvantages, such as inconvenience and contamination of disinfection by-products (e.g., chlorine disinfection). In this study, atmospheric surface plasma in argon mixed with air and plasma-activated water was found to efficiently inactivate bacteriophages, and plasma-activated water still had strong antiviral activity after prolonged storage. Furthermore, it was shown that bacteriophage inactivation was associated with damage to nucleic acids and proteins by singlet oxygen. An understanding of the biological effects of plasma-based treatment is useful to inform the development of plasma into a novel disinfecting strategy with convenience and no by-product.

## Abstract (precis)

Contamination with pathogenic and infectious viruses severely threatens human health and animal husbandry. Current methods for disinfection have different disadvantages, such as inconvenience and contamination of disinfection by-products (e.g., chlorine disinfection). In this study, atmospheric surface plasma in argon mixed with air and plasma-activated water was found to efficiently inactivate bacteriophages, and plasma-activated water still had strong antiviral activity after prolonged storage. Furthermore, it was shown that bacteriophage inactivation was associated with damage to nucleic acids and proteins by singlet oxygen. An understanding of the biological effects of plasma-based treatment is useful to inform the development of plasma into a novel disinfecting strategy with convenience and no by-product.

## INTRODUCTION

Contamination with microorganisms, especially pathogenic and infectious viruses, such as poliovirus and foot-and-mouth disease virus, is a threat to public health and animal husbandry. Current disinfection methods include UV irradiation and chemical disinfectants, with UV irradiation requiring a long processing time and chemical disinfectants leaving the by-product of contamination (1, 2). Thus, the development of efficient and safe disinfection strategies for these pathogenic microorganisms is of great significance for human health (3).

Cold atmospheric-pressure plasma (here, plasma) at or near room temperature generates numerous reactive oxygen species (ROS) and reactive nitrogen species (RNS), such as hydrogen peroxide (H_2_O_2_), singlet oxygen (^1^O_2_), ozone (O_3_), nitric oxide (˙NO), and hydroxyl radical (˙OH), as well as electrons, ions, and photons. These make plasma attractive for biomedical and environmental applications (4–9). Currently, plasma has been widely studied for bacterial inactivation and as therapy for infectious diseases (10, 11). Previous studies have reported that a form of plasma, dielectric barrier discharge (DBD), efficiently inactivated very small volumes (20 or 50 μl) of dry and wet Φ174 and λ bacteriophages in Tris-EDTA buffer by damaging the proteins and DNA of bacteriophages (12–14). Further, MS2 and feline calicivirus could also be inactivated by direct treatment of a plasma jet, the disadvantage of which was the limitation of the treatment area (15, 16). Recently, Su et al. used plasma-activated water, saline, and 0.3% H_2_O_2_ that were pretreated with a plasma jet to treat Newcastle disease virus and decreased its infectivity (17). Therefore, plasma and plasma-activated solutions have become potential alternative disinfectants.

Given the technical challenges and potential safety risk of working with pathogenic viruses, surrogate virus-bacteriophages are used to evaluate the antiviral activities of the plasma in this study. Three bacteriophages with different types of nucleic acids, T4 (double-stranded DNA), Φ174 (single-stranded DNA), and MS2 (RNA), were selected, and a surface discharge plasma was used in this study. Surface plasma or water activated by the surface plasma was used to treat water with bacteriophages. Our aim is to demonstrate the antiviral activities of the surface plasma and surface-plasma-activated water, as well as to unravel underlying disinfection mechanisms of the surface plasma and plasma-activated water.

## RESULTS AND DISCUSSION

### ROS and RNS generated by plasma.

**FIG 1:**
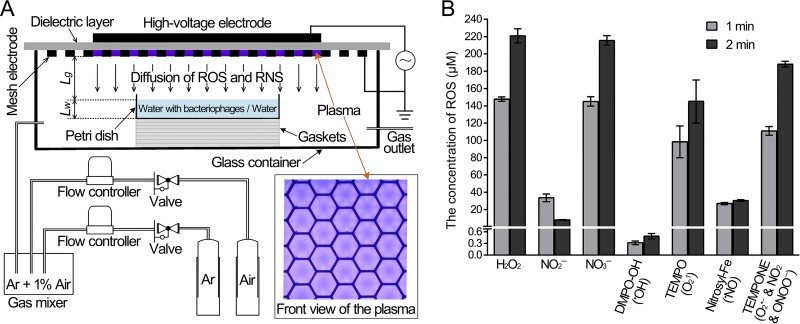
Reactive oxygen species (ROS) and reactive nitrogen species (RNS) generated by surface plasma. (A) Diagram of bacteriophage suspensions or water treated with surface plasma. (B) The concentrations of ROS, RNS, and spin trap in the water treated with plasma for 1 and 2 min.

Numerous types of gaseous ROS and RNS are generated by surface discharge, and some diffuse across the air gap (*Lg*) and then dissolve into the liquids. The plasma-induced aqueous ROS and RNS levels in the water were measured after plasma treatment for 1 and 2 min. We found that the long-lived species H_2_O_2_, NO_2_^−^, and NO_3_^−^, as well as the short-lived species ˙OH, ^1^O_2_, ˙NO, O_2_˙^−^, ˙NO_2_, and ONOO^−^ diffused into the water. The concentrations of aqueous H_2_O_2_, NO_2_^−^, and NO_3_^−^ after 2 min of plasma treatment were 221, 8, and 216 μM, respectively (Fig. 1B).

Short-lived species of ROS and RNS were identified and quantitated by electron spin resonance (ESR) spectroscopy by using four spin traps, 5,5-dimethyl-1-pyrroline *N*-oxide (DMPO) for trapping ˙OH, *N*-(dithiocarbamoyl)-*N*-methyl-d-glucamine (MGD) for trapping ˙NO, 2,2,6,6-tetramethylpiperidine (TEMP) for trapping ^1^O_2_, and 1-hydroxy-2,2,6,6-tetramethyl-4-oxo-piperidine (TEMPONE-H) for trapping O_2_˙^−^, ˙NO_2_, and ONOO^−^. The results were the concentrations of spin adducts, which only reflected the relative concentrations of the specific ROS and RNS. The concentrations of the spin adducts, DMPO-OH, 2,2,6,6-tetramethylpiperidine 1-oxyl (TEMPO), nitrosyl-Fe, and 4-oxo-2,2,6,6-tetramethylpiperidine-1-oxyl (TEMPONE) after a 2-min plasma treatment were 0.5, 145, 30, and 188 μM, respectively (Fig. 1B). These results indicated that the concentration of aqueous ˙OH should be very low, while that of aqueous ^1^O_2_ should be much higher. ROS and RNS generated by plasma are not a simple system but rather a chaotic system involved in numerous chemical reactions (18). ROS and RNS are widely believed to play a crucial role in plasma-induced biological effects (19).

### Inactivation of bacteriophages by plasma or plasma-activated water.

**FIG 2:**
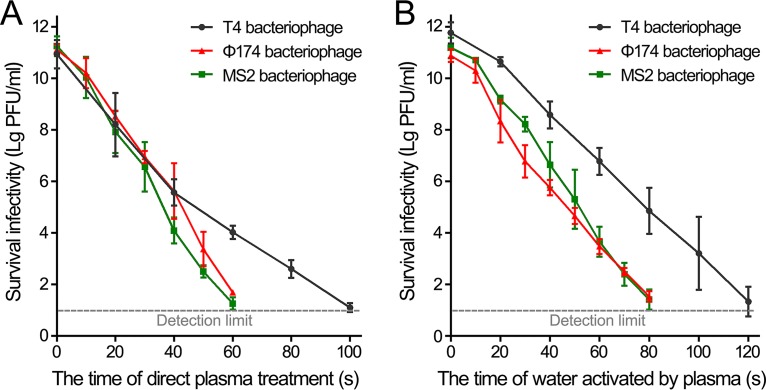
Inactivation of bacteriophages by plasma and plasma-activated water. (A) Direct plasma treatment. (B) Plasma-activated water treatment. Bacteriophage suspensions were directly treated with plasma or mixed with water that was treated with plasma, for the indicated times, and the treated samples were incubated for 1 h at 22°C. Surviving infectivity was quantified using serial dilution, plating, and counting of the resulting PFU. Data are representative of the results from three independent experiments. Error bars represent the standard deviation (SD).

To evaluate the abilities of plasma and plasma-activated water to inactivate bacteriophages, different bacteriophage suspensions were exposed to plasma or incubated with the plasma-activated water pretreated with plasma for various times, and the infectivity of treated bacteriophages was assayed by a determination of their residual PFU count. Direct treatment of the T4 bacteriophage with plasma for 40 s reduced the PFU from 8.7 × 10^10^ to 3.7 × 10^5^ PFU/ml, and 80 s of treatment gave a residual infectivity of approximately 400 PFU/ml, indicating that more than 99.99% phages lost their infectivity. Furthermore, treatment with plasma for 100 s completely abolished the infectivity of the T4 bacteriophage suspension (Fig. 2A). Treatment of the T4 bacteriophage with plasma-activated water that was pretreated with plasma for 60 s or 120 s for 1 h reduced the level from 5.8 × 10^11^ to 6.0 × 10^6^ and to approximately 20 PFU/ml, respectively (Fig. 2B), suggesting that the effective species could be stably maintained in the plasma-treated water. The same experiments with Φ174 and MS2 bacteriophages showed that after treatment with plasma for 30 s, the infectivities of Φ174 and MS2 bacteriophages were reduced by approximately 4.2 and 4.6 orders of magnitude, respectively, and both phages were almost completely inactivated after 60 s of treatment (Fig. 2A). These results indicated that Φ174 and MS2 bacteriophages are more sensitive to ROS and RNS generated by plasma than T4 bacteriophages. Then, the sensitivities of Φ174 and MS2 bacteriophages to plasma-activated water were tested by incubation of bacteriophage suspension and the water pretreated with plasma for 60 s; this revealed that the number of infective phages was reduced close to the detection limit for both phages (Fig. 2B). These results indicated that both plasma and plasma-activated water effectively inactivated the bacteriophages.

**FIG 3:**
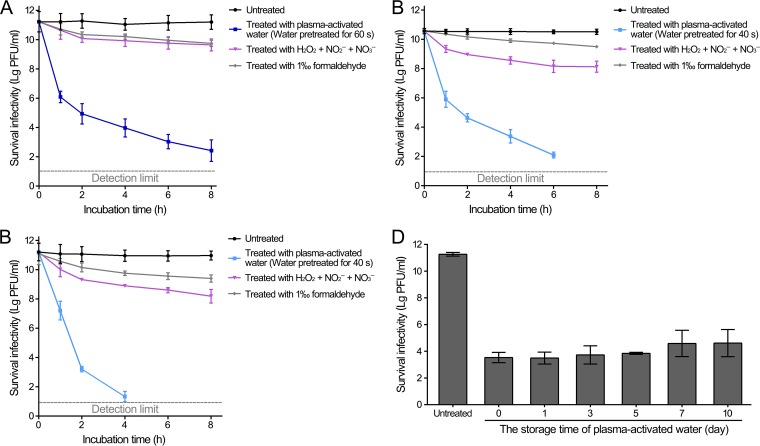
Comparative analysis of bacteriophages inactivated by plasma-activated water. (A) T4. (B) Φ174. (C) MS2. Bacteriophage suspensions were directly treated with water treated with plasma for 60 s or 40 s, and then 500 μM H_2_O_2_, 75 μM NO_2_^−^, and 500 μM NO_3_^−^ or 1% formaldehyde, and the treated samples were incubated at 22°C. Surviving infectivity was quantified at indicated time points using serial dilution, plating, and counting of the resulting PFU. (D) The storage of plasma-activated water. Plasma-activated water was stored at 22°C for indicated times. Then, the plasma-activated water was mixed with bacteriophage suspensions and incubated at 22°C for 1 h. Surviving infectivity was quantified using serial dilution and plating for PFU. Data are representative of the results from three independent experiments. Error bars represent the standard deviation (SD).

T4, Φ174, and MS2 bacteriophages were almost completely inactivated by plasma-activated water that was pretreated with plasma for 120 s (for T4) or 80 s (for Φ174 and MS2) (Fig. 2B). Then, water pretreated with plasma for half of the time, 60 s for T4 or 40 s for Φ174 and MS2, was incubated with bacteriophage suspensions, and the bacteriophage infectivity at different time points was measured (Fig. 3). After incubation for 4 and 8 h with plasma-activated water, the T4 bacteriophage was reduced approximately 7.2 and 8.8 orders of magnitude, respectively (Fig. 3A). The Φ174 and MS2 bacteriophages were reduced to 124 and 21 PFU/ml after incubation with plasma-activated water for 6 h and for 4 h, respectively (Fig. 3B and C). These results exhibited that the inactivation of bacteriophages by plasma-activated water was time dependent. Compared with the treatment of 1‰ formaldehyde, a reagent for virus inactivation, which only reduced the levels by less than 2 orders of magnitude after incubation for 8 h, the plasma-activated water was more effective (Fig. 3).

Then, the time of validity of the use of plasma-activated water was measured, and plasma-activated water stored at 22°C for 10 days exhibited slightly weaker antiviral activity than that of the freshly prepared plasma-activated water, probably due to the decay of reactive species during storage (Fig. 3D). The plasma-activated water stored in glass tubes did not exhibit differences in inactivating viruses from that stored in Eppendorf tubes, indicating that plasticware and glassware were both suitable for storing plasma-activated water (see Fig. S1 in the supplemental material). Moreover, exposure to light did not significantly undermine the antiviral activity of plasma-activated water, since there is no significant difference compared with that stored in the dark (Fig. S1). Despite its slightly reduced antiviral activity, the plasma-activated water was storable and transportable, demonstrating that the plasma-generated ROS and RNS might be used in both gaseous and aqueous forms, depending on the mode of application.

### Plasma caused aggregation of bacteriophages.

**FIG 4:**
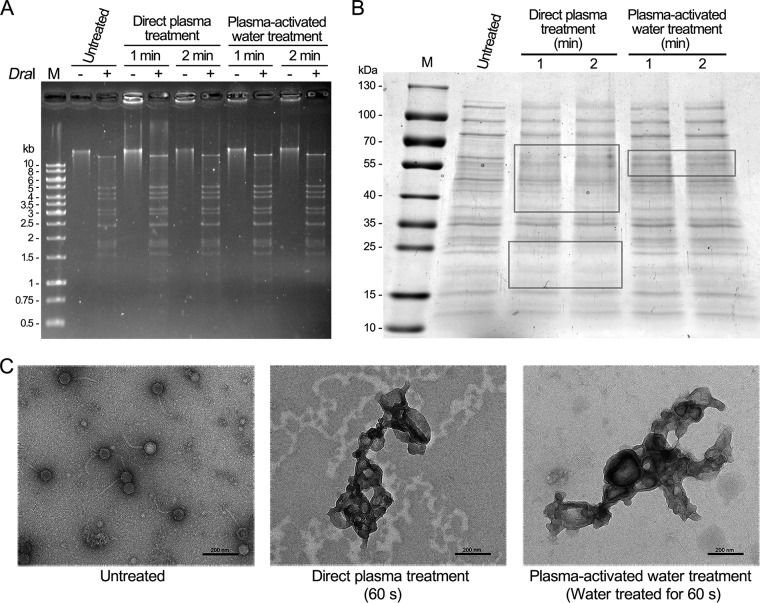
Analysis of T4 bacteriophages treated with plasma and plasma-activated water. (A) The genomic DNA of T4 bacteriophages. The genomic DNA of T4 bacteriophage treated with plasma, treated with plasma-activated water, or untreated, digested with DraI or undigested, was separated using 0.8% agarose gels and stained with ethidium bromide (EtBr). M, 1-kb DNA ladder. (B) The proteins of T4 bacteriophages. The proteins of T4 bacteriophage treated with plasma, treated with plasma-activated water, or untreated were analyzed by 6 to 15% gradient SDS-PAGE gels and stained with Coomassie blue R-250. M, molecular weight marker. (C) Transmission electron microscopy (TEM) analysis of T4 bacteriophages treated with plasma and plasma-activated water. The T4 bacteriophage treated with plasma, treated with plasma-activated water, or untreated were negative stained and examined using TEM.

Next, we investigated how the plasma could inactivate the bacteriophages. To this end, the DNA and proteins of T4 bacteriophage treated with plasma or plasma-activated water were analyzed. A large fraction of T4 genomic DNA was retained in the loading wells for T4 bacteriophage treated with plasma or plasma-activated water, while the DNA from untreated T4 bacteriophage exhibited a single band (Fig. 4A). However, after digestion with DraI, the signals in the sample wells were digested, and the DNA from the treated and untreated samples exhibited similar bands (Fig. 4A). The results suggested that T4 genomic DNAs could have been cross-linked themselves or with the coat proteins during plasma-based treatment, forming large DNA-protein complexes that cannot migrate during agarose gel electrophoresis. Compared with the untreated T4 bacteriophage, parts of the proteins from T4 bacteriophage treated with plasma or plasma-activated water slightly decreased, which probably was due to the degradation of proteins through oxidation (Fig. 4B, gray rectangles) (20). The damage induced by plasma-activated water was lesser than that induced by direct plasma treatment, which was likely because plasma-activated water lacked part of the species generated by plasma, such as some short-lived reactive species and UV (21). These results indicated that reactive species of plasma induced both DNA and protein damage to bacteriophages.

Further, the morphological changes in the T4 bacteriophage induced by plasma and plasma-activated water were investigated using transmission electron microscopy (TEM). The untreated T4 bacteriophages exhibited a typical structure, with an icosahedron head and tail (Fig. 4C). After plasma or plasma-activated water treatment, the T4 bacteriophages were severely aggregated and formed large complexes (Fig. 4C). The morphological study suggested that reactive species of plasma caused an interaction between adjacent bacteriophages, leading to the aggregation of T4 bacteriophages.

### Singlet oxygen played a primary role in bacteriophage inactivation.

**FIG 5:**
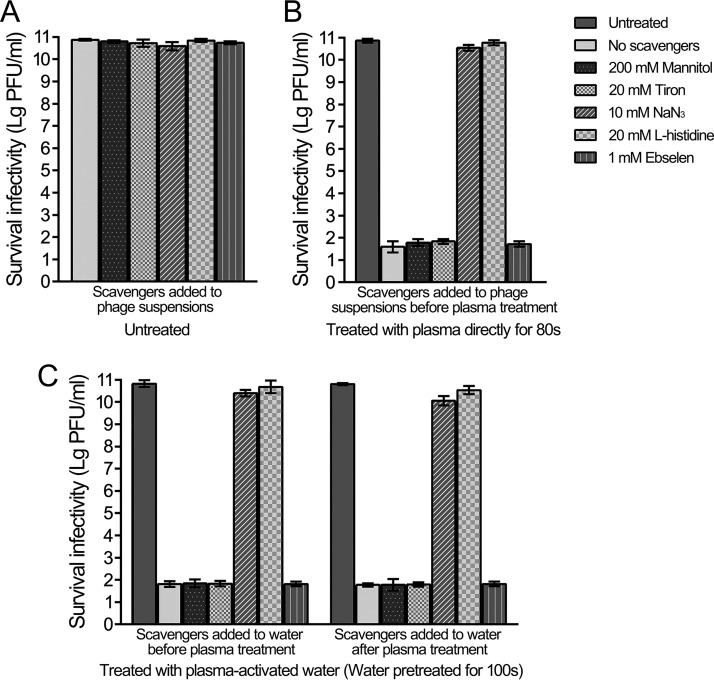
Singlet oxygen of plasma played a primary role in bacteriophage inactivation. (A) The scavengers for ROS and RNS did not evidently affect the infectivity of bacteriophages. ROS and RNS scavengers added to water with bacteriophages and incubated for 1 h at 22°C. (B) Direct plasma treatment. The ROS and RNS scavengers were added to bacteriophage suspensions, and then the bacteriophage suspensions were treated with plasma directly and incubated for 1 h at 22°C. (C) Plasma-activated water treatment. The ROS and RNS scavengers were added to the water before or after the plasma treatment. Then, the plasma-activated water in the presence and absence of scavengers was incubated with bacteriophage suspensions and kept for 1 h at 22°C. Surviving infectivity was quantified by serial dilution, plating, and counting of the resulting PFU. Data are representative of the results from three independent experiments. Error bars represent the standard deviation (SD).

Plasma-treated solutions contain numerous active species, such as the long-lived species H_2_O_2_, NO_2_^−^, and NO_3_^−^, as well as the short-lived species ˙OH, ^1^O_2_, ˙NO, O_2_˙^−^, ˙NO_2_, and ONOO^−^ (Fig. 1B). To evaluate the effect of long-lived species, a mixture of H_2_O_2_ (500 μM), NO_2_^−^ (75 μM), and NO_3_^−^ (500 μM) was used to treat the bacteriophages. However, this only reduced the levels by 1.6 to 3 orders of magnitude, indicating that the three long-lived species are not the main functional factors (Fig. 3). Hence, short-lived species should be considered more important. The ROS and RNS scavengers, which were mannitol for ˙OH, tiron for O_2_˙^−^, sodium azide and l-histidine for ^1^O_2_, and ebselen for ONOO^−^, were used to distinguish the role of the different short-lived species, and the scavengers did not obviously affect bacteriophage infectivity (Fig. 5A). Sodium azide and l-histidine almost entirely eliminated the inactivation effects of direct plasma treatment, whereas other ROS and RNS scavengers exhibited nonsignificant effects (Fig. 5B). The effects of plasma-activated water were also eliminated by sodium azide and l-histidine, which were added to the water both before and after plasma treatment and were not eliminated by other ROS and RNS scavengers (Fig. 5C). Singlet oxygen was detected in both water treated with plasma directly and plasma-activated water using a highly selective probe of singlet oxygen–*trans*-1-(2′-methoxyvinyl)pyrene (tMVP) (Fig. S2). These data suggest that singlet oxygen was the main functional species of the plasma and plasma-activated water in the inactivation of bacteriophages.

Singlet oxygen is highly active and readily reacts with various biological molecules, including DNA and proteins (22–24). Singlet oxygen rapidly reacts with cysteine to generate the major product of cystine (R-cys-S-S-cys-R) with disulfides, which could result in the interaction and aggregation of bacteriophages (20). Singlet oxygen also selectively reacted with tyrosine, tryptophan, and histidine to produce hydroperoxides, which could inactivate enzyme activities (20). For DNA, singlet oxygen could oxidize guanine and induce cross-links between guanine and lysine, which would be responsible for the large complexes formed by T4 genomic DNA (25). The bacteriophage inactivation by singlet oxygen generated in UV-illuminated fullerol similarly induced the cross-linking of capsid proteins, which were the probable cause of phage inactivation (26). The inactivation of bacteriophages by plasma was mainly mediated by the multiple effects of singlet oxygen.

Plasma-based treatment provides an effective strategy for environmental disinfection in spite of some limitations. Singlet oxygen could also react with a wide range of organic compounds, such as olefins and phenols (27). The presence of organic compounds would inevitably reduce the inactivation abilities of plasma and plasma-activated water. Therefore, plasma-based disinfectants are more applicable to space and water disinfection.

### Conclusions.

**FIG 6:**
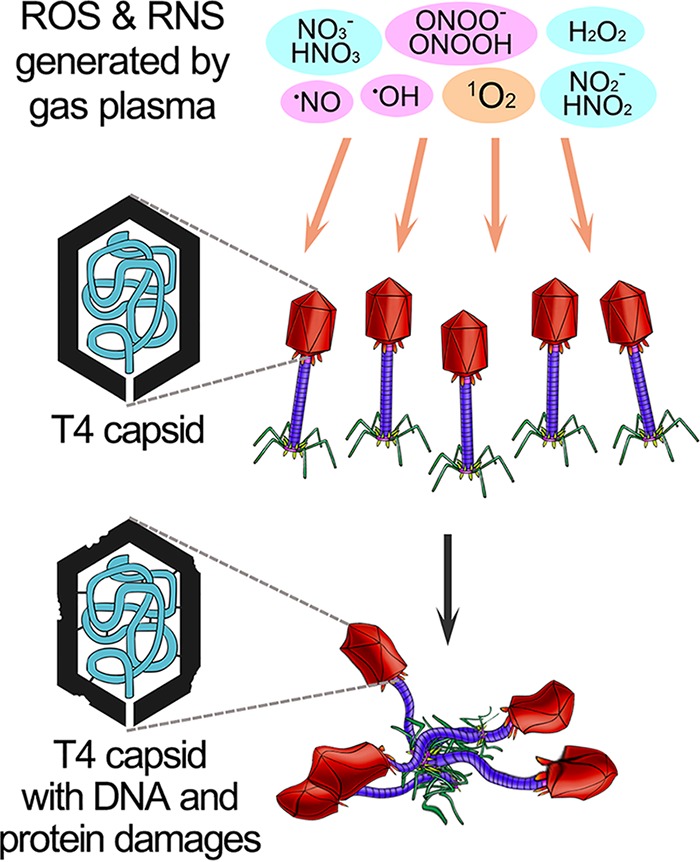
The inactivation of bacteriophage T4 by ROS and RNS of plasma.

Based on these results, a model of bacteriophage inactivation by plasma and plasma-activated water was proposed (Fig. 6). Plasma-generated reactive species, especially singlet oxygen, efficiently inactivated different kinds of bacteriophages in water, including double-stranded DNA, single-stranded DNA, and RNA bacteriophages, by damaging both nucleic acid and proteins and causing aggregation of the bacteriophages. It is useful for the design and improvement of novel plasma devices with potential application for understanding the biological and chemical mechanisms of virus inactivation by plasma-based treatment. Compared with ROS derived from inorganic or organic chemicals, ROS generated in plasma was a direct additive, so it would not bring chemical residual contamination after the treatment process. Plasma-based treatment efficiently inactivated different classes of viruses and could be explored as a novel strategy for disinfection to combat environmental problems caused by viruses.

## MATERIALS AND METHODS

### Plasma device and water treatments.

The surface discharge device consisted of a plane high-voltage electrode, a liquid-facing grounded mesh electrode, and a dielectric layer (made of polytetrafluoroethylene) sandwiched between the two electrodes (Fig. 1A). The surface plasma is generated in the mesh elements of the grounded electrode when a sinusoidal high voltage is applied, and the discharge power density was maintained at 0.2 W/cm^2^ for this study. As shown in Fig. 1A, each mesh element has a hexagonal shape, and the plasma has good mesh-to-mesh homogeneity. The petri dish had dimensions of 11 cm long by 7 cm wide, which were much smaller than those of the surface plasma (12 cm by 8 cm). The bacteriophage suspensions or water (8 ml) in the petri dish with the depth (*Lw*) of about 1 mm was placed underneath the plasma, while the air gap (*Lg*) between the plasma and the liquid surface was 8 mm. The surface air plasma and the bacteriophage suspension were well sealed in an organic glass box. A gas mixture of argon and artificial air (79% N_2_ plus 21% O_2_) was allowed to flow through the box at a constant rate of 4 liters/min, and the volume ratio of artificial air was controlled at 1%. Compared to the surface discharge in air, the addition of argon enhanced the production efficiency of the reactive species and their fluxes underneath the bacteriophage suspension or water by diffusion. For more detail of the surface discharge reactor, please refer to our previous reports (18, 28).

### Measurement of aqueous ROS and RNS generated by plasma.

The concentrations of H_2_O_2_ and NO_2_^−^/NO_3_^−^ in the water were measured using a hydrogen peroxide/peroxidase assay kit (Thermo Fisher Scientific) and a nitrite/nitrate colorimetric assay kit (Cayman), respectively. ˙OH, ^1^O_2_, ˙NO, O_2_˙^−^, ˙NO_2_, and ONOO^−^ were measured using an electron spin resonance (ESR) spectroscope (Bruker) with relevant spin traps (29). The spin traps were 100 mM 5,5-dimethyl-1-pyrroline *N*-oxide (DMPO; Dojindo) for trapping ˙OH, 5 mM *N*-(dithiocarbamoyl)-*N*-methyl-d-glucamine (MGD; Dojindo) for trapping ˙NO, 10 mM 2,2,6,6-tetramethylpiperidine (TEMP; TCI) for trapping ^1^O_2_, and 10 mM 1-hydroxy-2,2,6,6-tetramethyl-4-oxo-piperidine (TEMPONE-H; Enzo) for trapping O_2_˙^−^, ˙NO_2_, and ONOO^−^.

### Bacteriophage propagation and inactivation assay.

Bacteriophages T4 (provided by Xiaoqin Lai, Institute of Microbiology, Chinese Academy of Sciences), Φ174 (provided by Yigang Tong, Beijing Institute of Microbiology and Epidemiology), and MS2 (ATCC 15597-B1) and their host strain Escherichia coli JM109 (ATCC 53323) were used. The phages (1 ml) were used to infect an early stationary-phase culture of the host strain (100 ml), and infected cultures were then propagated at 37°C for 3 to 5 h. Phage suspensions were then prepared with the infected cultures by centrifugation at 3,000 × *g* for 10 min to remove the bacterial cells and debris and by filtration with 0.22-μm syringe filters (Millipore). The filtered supernatants were dialyzed with water using a concentrator (10-kDa molecular weight cutoff; Millipore), yielding stocks of phage suspension with a titer of 10^11^ to 10^12^. The stocks were stored at 4°C and investigated in 2 weeks.

Bacteriophage suspensions were treated with plasma for increasing times, plasma-activated water (volume ratio, 1:1) that was pretreated with plasma for various times, 500 μM H_2_O_2_, 75 μM NO_2_^−^, and 500 μM NO_3_^−^ or 1% formaldehyde, and then incubated at 22°C for 1 h or the times indicated in Fig. 3A, B, and C, and the bacteriophage titers in the water were measured using the double-agar-layer method. The early stationary-phase host strain was collected by centrifugation, washed once with 10 mM magnesium sulfate (MgSO_4_), and suspended in 10 mM MgSO_4_. After treatment, 100 μl of phage was incubated with 200 μl host strain suspension at 37°C for 20 min. Then, the incubated solutions were mixed with soft agar (LB broth with 0.7% agar) that was preheated to 45°C and plated onto the bottom layer of the agar (LB broth with 1.5% agar). The plates were cultured at 37°C overnight, and the plaques were subsequently counted.

### Bacteriophage inactivation assay of the plasma-activated water after storage.

The water was treated with plasma for 100 s and stored in 1.5-ml Eppendorf tubes made of polypropylene or glass tubes at 22°C in the dark or in the light for different days, as indicated in Fig. 3D. After storage, the plasma-activated water was mixed with T4 bacteriophage suspensions (volume ratio, 1:1) and incubated at 22°C for 1 h. Then, the bacteriophage titers in the water were measured using the double-agar-layer method, as described previously.

### Analysis of bacteriophage DNA.

Phage genomic DNA was extracted from treated or untreated T4 bacteriophage samples using a viral DNA kit (Omega). Then, the T4 DNA samples were digested with DraI (TaKaRa) and separated using 0.8% agarose gels in 0.5× Tris-borate-EDTA (TBE) buffer at 50 V for 3 to 4 h. The gels were stained with ethidium bromide (EtBr) and then examined and photographed using a BioDoc-It imaging system (UVP).

### SDS-PAGE.

T4 bacteriophages that were treated with plasma or plasma-activated water or were untreated (20 μl) were mixed with Laemmli buffer (Bio-Rad) and analyzed using 6 to 15% gradient SDS-PAGE gels. The gels were stained with Coomassie blue R-250 and scanned using a GS-800 calibrated densitometer (Bio-Rad).

### Transmission electron microscopy.

T4 bacteriophages that were treated with plasma or plasma-activated water or were untreated were dropped onto carbon-coated grids and kept for 10 min at 22°C. Then, the excess liquid was discarded, and the grids were covered with 1% uranyl acetate for 30 s at 22°C. These stained samples were examined using an FEI Talos F200C transmission electron microscope operating at 200 kV at ×28,000 magnification.

### Analysis of ROS and RNS scavengers.

Chemical scavengers for ROS and RNS, mannitol (MP Biomedicals), tiron (Sigma-Aldrich), sodium azide (Sigma-Aldrich), l-histidine (Sigma-Aldrich), and ebselen (TCI), were used. For direct plasma treatment, a final concentration of 200 mM mannitol, 20 mM tiron, 10 mM sodium azide, 10 mM l-histidine, or 1 mM ebselen was added to the water containing bacteriophages. Then, the samples with or without scavengers were treated with plasma for 2 min and incubated at 22°C for 1 h. For the plasma-activated water treatment, a final concentration of 200 mM mannitol, 20 mM tiron, 10 mM sodium azide, 10 mM l-histidine, or 1 mM ebselen was added to water before or after plasma treatment for 2 min. Then, the water with or without scavengers was incubated with an equal volume of water containing bacteriophages at 22°C for 1 h. The inactivation rates were examined as described above.

### Statistical analysis.

All experiments were performed independently at least three times. Statistical analysis was performed using GraphPad Prism version 5. Statistical analyses were performed in SPSS 13.0 (IBM, Armonk, NY, USA) using the *t* test and Kruskal-Wallis test. The statistical significance of the data was established at a *P* value of <0.05.

## Supplementary Material

Supplemental file 1
