# Supplementary material for: Mechanism of Virus Inactivation by Cold Atmospheric-Pressure Plasma and Plasma-Activated Water
Source: Appl Environ Microbiol. 2018 Aug 17;84(17):e00726-18. doi: 10.1128/AEM.00726-18 (PMC6102979; doi:10.1128/AEM.00726-18)

Supplemental Materials

Figure S1. The effects of storage conditions on plasma-activated water. Plasma-activated water was stored in eppendorf tubes or glass tubes in the dark or light at 22°C for indicated times. Then the plasma-activated water were mixed with bacteriophage suspensions and incubated at 22°C for 1 h. Surviving infectivity was quantified using serial dilution and plating for PFU. Data are representative of three independent experiments. Error bars represent standard deviation (SD).

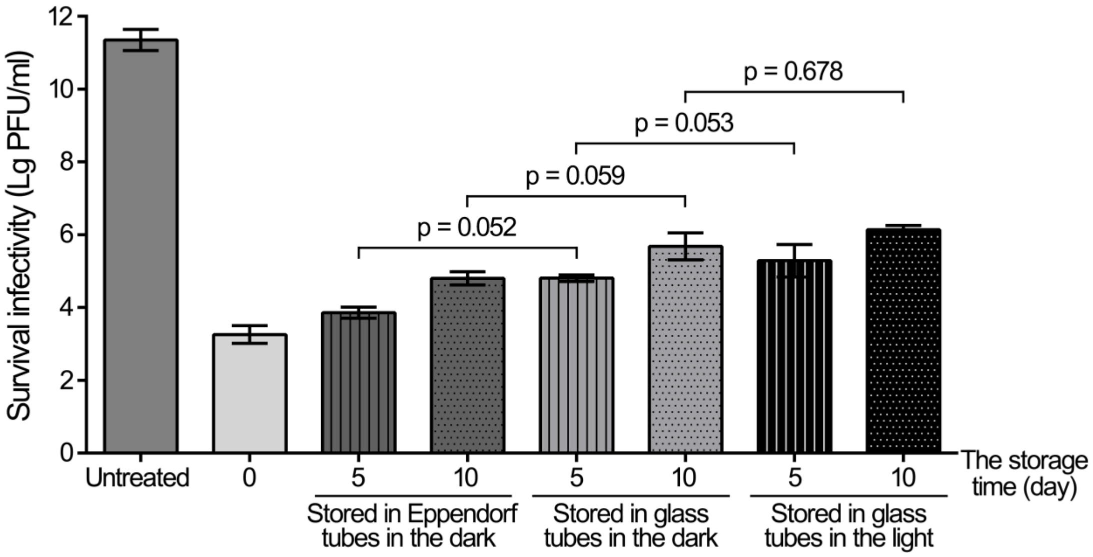

Figure S2. Measurements of singlet oxygen in water treated with plasma directly and plasma-activated water. The probe trans-1-(2'-methoxyvinyl)pyrene (tMVP, J&K Scientific) was used to detect the singlet oxygen. For plasma direct treatment, tMVP was added to water in absence and presence of NaN_3 (10 mM) or histidine (10 mM) at a final concentration of 40 μM and treated with plasma for 2 min. For plasma-activated water, water was treated with plasma for 2 min, and NaN_3 (10 mM) or histidine (10 mM) was incubated with plasma-activated water. Then, tMVP was added to plasma-activated water in absence and presence of NaN_3 or histidine at a final concentration of 40 μM. The fluorescence intensities were detected using a microplate reader (Thermo Scientific Varioskan Flash) at the excitation and emission wavelengths (405/460 nm).

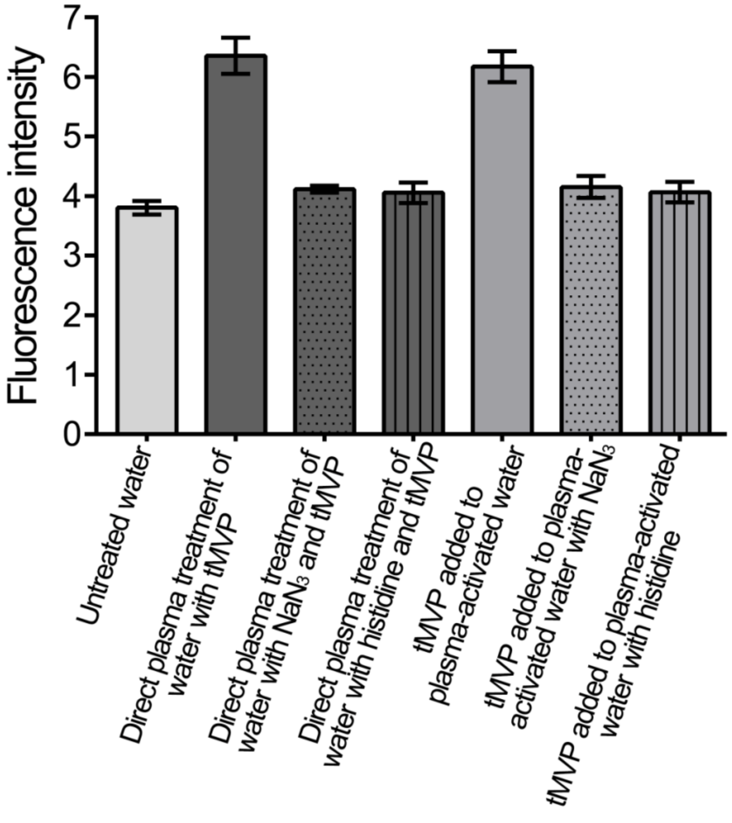

Supplement: Supplemental file 1 [file zam017188690s1.pdf]
